# Non-Invasive Imaging of Normalized Solid Stress in Cancers in Vivo

**DOI:** 10.1109/JTEHM.2019.2932059

**Published:** 2019-09-13

**Authors:** Md Tauhidul Islam, Ennio Tasciotti, Raffaella Righetti

**Affiliations:** 1 Department of Electrical and Computer EngineeringTexas A&M UniversityCollege StationTX77843USA; 2 Center of Biomimetic MedicineHouston Methodist Research Institute 167626 HoustonTX77030USA

**Keywords:** Cancer imaging, elastography, microenvironment, poroelastography, solid stress, interstitial fluid pressure, cancer biomechanics, oncophysics

## Abstract

The solid stress (SSg) that develops inside a cancer is an important marker of cancer’s growth, invasion and metastasis. Currently, there are no non-invasive methods to image SSg inside tumors. In this paper, we develop a new, non-invasive and cost-effective imaging method to assess SSg inside tumors that uses ultrasound poroelastography. Center to the proposed method is a novel analytical model, which demonstrates that SSg and the compression-induced stress (SSc) that generates inside the cancer in a poroelastography experiment have the same spatial distribution. To show the clinical feasibility of the proposed technique, we imaged and analyzed the normalized SSg inside treated and untreated human breast cancers in a small animal model. Given the clinical significance of assessing SSg in cancers and the advantages of the proposed ultrasonic methods, our technique could have a great impact on cancer diagnosis, prognosis and treatment methods.

## Graphical abstract

We present a new, non-invasive and cost-effective imaging method to assess solid stress inside tumors using ultrasound poroelastography. To show the clinical feasibility of the proposed technique, we imaged and analyzed the normalized solid stress inside treated and untreated human breast cancers in a small animal model. Given the clinical significance of assessing solid stress in cancers and the advantages of the proposed ultrasonic methods, our technique can have a great impact on cancer diagnosis, prognosis and treatment.

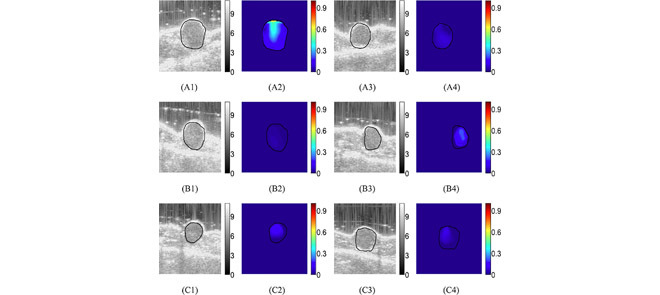

## I. Introduction

The mechanical microenvironment plays an important role in the growth, invasion and metastasis of cancers [1]–[4]. The solid stress (SSg) that develops inside a cancer is an important component of the mechanical microenvironment and an influential factor in cancer’s growth and metastasis. The proof of existence of SSg inside tumors is relatively recent and has originated from the discovery that blood and lymphatic vessels inside the tumor are mechanically compressed [5]–[7].

There are three main sources of SSg inside cancers: external stress, swelling stress and growth-induced or residual stress. The external stress is created by the resistance of the normal tissue to newly grown cells in cancers, which try to expand against the surrounding normal tissue. The surrounding tissue resists this expansion by exerting an opposite stress. Swelling stress is caused by chemical expansion as the interstitial space of the cancer may have a high concentration of negatively charged hyaluronan chains, and the repulsive electrostatic force among these negative charges can cause swelling of the tumor. The residual stress can be defined as the remaining stress inside a body, when all the external loads on the body have been removed [8].

Assessment of SSg inside tumors is of great clinical significance for various reasons. By compressing the blood vessels inside the tumor, SSg can cause deficiency of oxygen supply in the cancer, also known as hypoxia. Hypoxia is responsible for impaired perfusion and can reduce the killing potential of the immune cells [9]. SSg also affects the growth of cancerous cells [10] and promotes their collective migration [11]. Finally, compression of blood and lymphatic vessels due to SSg creates unfavorable environment for targeted drug delivery in two ways. First, the compression of blood vessels results in reduced perfusion and lower amount of drugs that can reach the central portion of the tumor [12]. Second, the compression and collapse of lymphatic vessels reduce the outflow of interstitial fluid from the tumor, which, in turn, increases the interstitial fluid pressure (IFP) inside the tumor. This elevation of IFP inside the tumor reduces the amount of drug delivered to the tumor and induces tumor progression [13].

A number of invasive techniques have been proposed for estimating SSg inside cancers. In the works of Stylianopoulos *et al.* [14] and Nia *et al.* [15], the authors proposed multiple invasive techniques based on incision of the tumor. The techniques based on partial cut of the tumor developed by Stylianopoulos *et al.* [14] are limited to bulk estimation of the stress. Nia *et al.* [15] developed several methods, such as planar cut, slicing and needle-biopsy, to measure SSg. These methods are based on the idea of releasing the stress in a controlled way and measuring the stress-induced deformation in the cancer via high resolution ultrasound or optical microscope. The main drawback of the aforementioned techniques is their invasive nature, which limits their applicability in vivo. Another limitation is that they provide only an average measurement of SSg.

Ultrasound elastography (USE) is a non-invasive, safe and cost-effective imaging modality that is used to assess the strains generated in the tissue by the application of a small external compression [16], [17]. Poroelastography is a branch of elastography, where the tissue is assumed to behave as a poroelastic material consisting of solid and fluid phases [18], [19]. The main motivation behind poroelastography is that many diseases, such as cancer, lymphedema etc., affect the fluid pressure gradients that generate in the tissue during a poroelastography experiment [20].

In a recent work from our group [21], we proved that the solid stress inside a tumor and the solid stress generated in a poroelastography experiment have the same spatial distribution. Based on this theory, we propose a new ultrasound poroelastography technique to determine the normalized SSg, which we refer to as “SSn” inside cancers. We first develop an analytical model of the SSg inside tumors based on the findings reported in [21]. We then use this analytical model to obtain an analytical expression of the SSn inside tumors. To show the clinical feasibility of the proposed method, SSn is imaged in treated and untreated breast cancers in vivo, and the results are statistically analyzed.

## II. Methods

### A. Analytical Model for Radial and Circumferential Solid Stress

#### 1) Assumptions

Our analytical model for radial and circumferential SSc inside the tumor is based on the following assumptions: 1) the tumor and normal tissues behave as poroelastic materials, and biphasic theories can be applied to describe their behavior; 2) the tumor is spherical in shape; 3) the tumor is perfectly bonded to the normal tissues; 4) the spatial distribution of the mechanical properties inside the tumor is uniform; 5) the assumption of ‘remote load’ is satisfied inside the tumor, i.e., the tumor is much smaller in comparison to the sample [22]; and 6) the mechanical properties (i.e., Young’s modulus, Poisson’s ratio, vascular permeability, interstitial permeability etc.) of cancers and normal tissues are uniform [23]–[25].

#### 2) Theory

**FIGURE 1.**
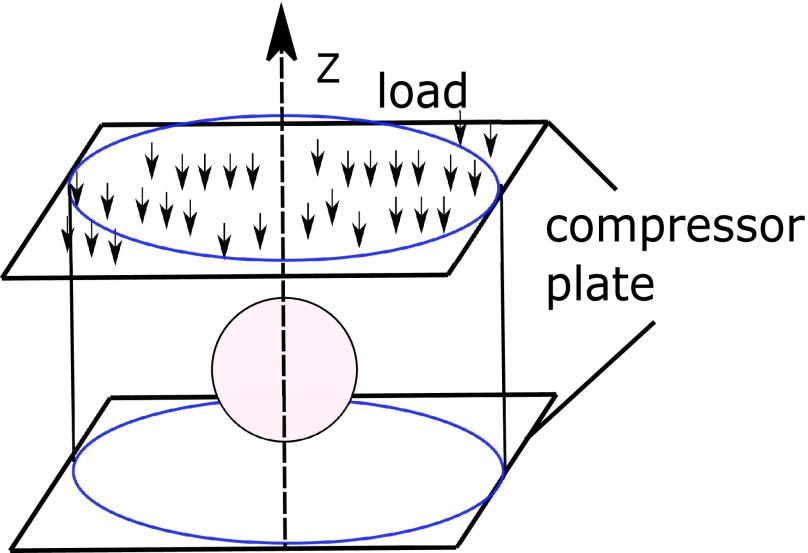
A schematic of a cylindrical sample of a poroelastic material with a spherical poroelastic inclusion of radius $a$. The sample is compressed between two impermeable plates. The compression is applied along the negative $z$ direction.

The proposed analytical model for SSg inside cancers is based on a spherical model of the tumor as shown in Fig. 1. Fig. 1 shows an inclusion (representing a tumor) embedded inside a cylindrical background (representing normal tissue). The protocol for applying compression in a poroelastography experiment is also shown in this figure.

The equation for the compression-induced fluid pressure (FPc) inside a spherical tumor in a poroelastography experiment at a time $t_{0}$ can be written as [26]

\begin{equation*} p(R)=\Psi \left({1-\frac {\sinh {\left({\alpha \frac {R}{a}}\right)}}{\frac {R}{a}\sinh {\alpha }}}\right),\quad \text {where}~\alpha =a \sqrt {\frac {L_{p}}{k}\frac {S}{V}}.\qquad \tag{1}\end{equation*}

Here, $\Psi =\frac {W}{\alpha ^{2}}$ is a constant related to the peak of p(R), $P_{0}$. $P_{0}=\Psi (1-\alpha \,\, \text {cosech} (\alpha))$ where $\alpha $ is the spatial distribution parameter of IFP and $a$ is the radius of the tumor. $L_{p}$ and $k$ are the vascular permeability and interstitial permeability of the tumor, respectively. $\frac {S}{V}$ is the surface area to volume ratio of the capillary walls inside the tumor.

The relationship between the radial/circumferential SSc and FPc can be written as [21]

\begin{align*} \sigma ^{c}_{RR} (R,t)=&\sigma _{RR}^{a}-p(R,t), \tag{2}\\ \sigma ^{c}_{\theta \theta }(R,t)=&\sigma _{\theta \theta }^{a}-p(R,t), \tag{3}\end{align*}

where $\sigma _{RR}^{a}$ and $\sigma _{\theta \theta }^{a}$ are the radial SSc and circumferential SSc at the tumor boundary, respectively. $\sigma _{RR}^{a}$ and $\sigma _{\theta \theta }^{a}$ can be computed using Eshelby’s theory by knowledge of the applied stress, geometry of the tumor and Young’s modulus and Poisson’s ratio of the tumor and background normal tissue [22]. In elastography experiments, we normally assume the axisymmetry of the material and strain components, which allows us to use cylindrical coordinates for analysis and presentation of strain and stress data [27]–[30]. The formulas for computation of the stress components in spherical coordinates from cylindrical coordinates can be found in Appendix A.

In [21], we theoretically demonstrated (and validated with simulations) that the solid stress due to tumor growth, SSg, and the poroelastography compression-induced solid stress, SSc, have the same spatial distributions (see Appendix B for proof). Thus, the equations for the radial SSg and the circumferential SSg can be written as

\begin{align*} \sigma ^{g}_{RR}(R)=&-\Omega _{bR}-\Omega _{pR} \left({1-\frac {\sinh {\left({\alpha \frac {R}{a}}\right)}}{\frac {R}{a}\sinh {\alpha }}}\right), \tag{4}\\ \sigma ^{g}_{\theta \theta }(R)=&-\Omega _{bT}-\Omega _{pT} \left({1-\frac {\sinh {\left({\alpha \frac {R}{a}}\right)}}{\frac {R}{a}\sinh {\alpha }}}\right). \tag{5}\end{align*}

Here, $\Omega _{bR}$ and $\Omega _{bT}$ are the values of radial SSg and circumferential SSg at the tumor’s boundary, respectively. $\Omega _{pR}$ is related to the peak radial SSg $S_{R}$, as $S_{R}=-\Omega _{bR}-\Omega _{pR}(1-\alpha \,\, \text {cosech} (\alpha))$ and $\Omega _{pT}$ is related to the peak circumferential SSg $S_{T}$ as $S_{T}=-\Omega _{bT}-\Omega _{pT}(1-\alpha \,\, \text {cosech} (\alpha))$. Therefore, the normalized SSg, SSn, can be expressed as

\begin{align*}&\hspace {-1.5pc}SS_{n} (R) \\=&\frac {\sigma ^{g}_{RR}(R)+\Omega _{bR}}{-\Omega _{pR}}=\frac {\sigma ^{g}_{\theta \theta }(R)+\Omega _{bT}}{-\Omega _{pT}} \\=&1-\frac {\sinh {\left({\alpha \frac {R}{a}}\right)}}{\frac {R}{a}\sinh {\alpha }}=\frac {\sigma ^{c}_{RR}(R)-\sigma ^{a}_{RR}}{-\Psi }=\frac {\sigma ^{c}_{\theta \theta }(R)-\sigma ^{a}_{\theta \theta }}{-\Psi }.\tag{6}\end{align*}

The peak value of SSn, $SS_{n,p}$ can be written as

\begin{equation*} SS_{n,p}=\frac {S_{R}+\Omega _{bR}}{-\Omega _{pR}}=\frac {S_{T}+\Omega _{bT}}{-\Omega _{pT}}=1-\alpha ~ \text {cosech} (\alpha).\qquad \tag{7}\end{equation*}

### B. In Vivo Experiments

Twelve mice (6 untreated, 6 treated) implanted with triple negative breast cancer (TNBC) were scanned once a week for three subsequent weeks. A mouse model of human TNBC was used for these experiments. TNBC patient derived xenograft (PDX) tumors was established by placing small pieces of freshly collected TNBC human cancer xenografts (BCM-4913) derived from primary human TNBC in the fat pad of immunocompromised female NOD/SCID gamma (NSG) mice [31]. In vivo experiments were approved by the Houston Methodist Research Institute, Institutional Animal Care and Use Committee (ACUC-approved protocol # AUP-0614-0033). Mice were kept untreated (n = 6) or were treated with epirubicin (n = 3) and LEPILOX (liposomes loaded with Epirubicin and conjugated with a targeting anti-LOX antibody on the particle surface, n = 3) for three weeks. The dose of each drug was 3 mg/kg body weight once a week. Each poroelastography experimental session was 5 m long, during which 3–4 RF data acquisitions (of duration 1 m each) were obtained.

The samples were scanned using a 38-mm real-time Sonix RP linear-array scanner (Ultrasonix, Richmond, BC, Canada) that has 128 elements, a bandwidth between 5 and 14 MHz, a center frequency of 6.6 MHz, 50% fractional bandwidth at −6 dB, sampling frequency of 40 MHz, and 1 mm beamwidth at the focus. A force sensor (Tekscan FlexiForce) was placed between the top surface of the gel pad and the compressor plate to record the applied force during compression. In all our poroelastography experiments, creep compression was performed in the region of interest in the animals. In a creep compression protocol, a constant pressure is applied on the sample. In our experiments, we applied a uniaxial pressure of 1–4 kPa for one minute [27].

To compute the elastograms from the pre- and post-compressed RF data in simulations and experiments, we used the method described in [32] and [33]. The method in [32] is a two-step method, which uses dynamic programming elastography (DPE) and Horn-Schunck optical flow estimation (HS). In our study, to compute the axial and lateral displacements with the DPE, the range of variation of axial displacement was set to 0 to −60 data points, and the range of variation of lateral displacement was set to −4 to 4 data points. The values of regularization weights along the axial and lateral directions were set to 0.15. For estimating the displacements by HS, the trade-off parameter $\beta $ was assumed to be 1. The number of pyramid levels was assumed as 4, and the maximum number of warping per pyramid level was set to 3. To warp and up-scale from coarse to fine scales, bi-cubic interpolation was used on the pre- and post-compression RF data. For the filtering technique [33], the length of the Kalman window ($W_{k}$) was taken as 13 for both axial and lateral strains estimation. The value of $\theta $ was set to $\frac {\pi }{30}$. $k_{max}$ and $k_{min}$ were set to 28 and 2. The value of $N$ was set to 3 and the value of $\sigma $ to 10. The value of $b$ was taken as 4 and $a$ was taken as 0.25.

The axial and lateral strains at a specific time point were calculated in a cumulative manner [34]. The SSn images reported in this paper are those corresponding to the 10 s time point calculated using $SS_{n}(R)=\frac {\sigma ^{c}_{RR}(R)-\sigma ^{a}_{RR}}{-\Psi }$. $\sigma ^{c}_{RR}$ and $\Psi $ were calculated using the theories developed in [21], [26]. The borders of the cancers were segmented on the in vivo axial strain elastograms [27], as these borders are not always evident in the B-mode images. The Young’s modulus and Poisson’s ratio of tumors and normal tissues were computed using the method described in [27].

To determine the value of $\alpha $ in vivo, we used the method described in Appendix C [21]. $SS_{n,p}$ was computed using the estimated $\alpha $ in eq. 7. Statistical significance between the treated and untreated in vivo results was determined using the Kruskal-Wallis test implemented using Matlab (MathWorks Inc., Natick, MA, USA).

## III. Results

**FIGURE 2.**
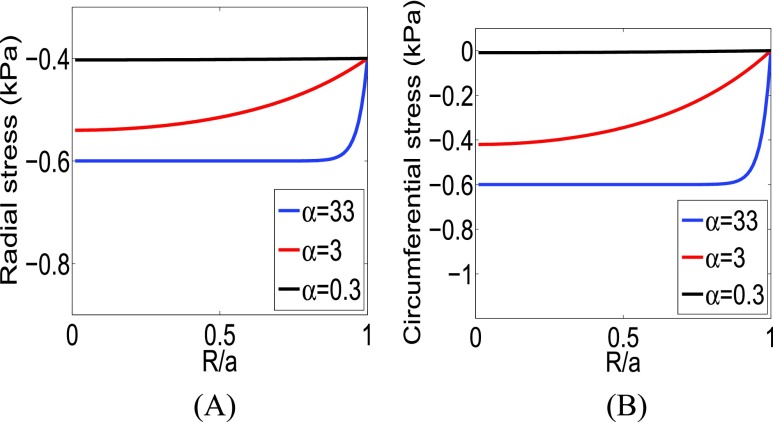
(A) Radial SSg and (B) circumferential SSg from the analytical model for $\alpha =33$, $\alpha =3$ and $\alpha =0.3$.

Analytical Model:

The behavior of the radial and circumferential SSg as a function of $\alpha $ as predicted by the analytical model is shown in Fig. 2. The radial and circumferential SSg are computed from eqs. (15) and (16) in Fig. 2. We used $\Omega _{bR}=0.4\,\,\text {kPa}, \Omega _{pR}=0.2\,\,\text {kPa}, \Omega _{bT}=0\,\,\text {kPa}, \Omega _{pT}=0.6\,\,\text {kPa}$ and $\alpha =33$, $\alpha =3$ and $\alpha =0.3$ in eqs. (15) and (16), respectively [1]–[3], [35]. From Fig. 2, we see that the radial and circumferential SSg have highest value at the center of the inclusion (tumor) and reduce gradually towards the periphery of the inclusion. Moreover, from Fig. 2, we see that the absolute peak values of the radial and circumferential SSg depend on the value of $\alpha $, i.e., peak stresses increase/decrease as $\alpha $ increases/decreases.

**FIGURE 3.**
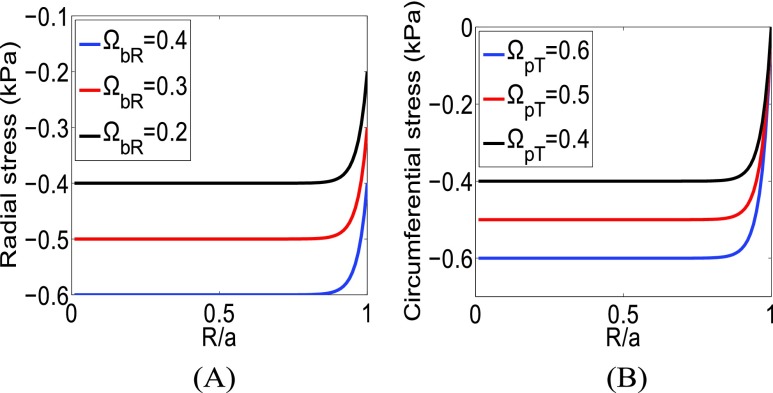
(A) Radial SSg from the analytical model for $\Omega _{bR}=0.2$, $\Omega _{bR}=0.3$ and $\Omega _{bR}=0.4$. (B) Circumferential SSg from the analytical model for $\Omega _{pT}=0.4$, $\Omega _{pT}=0.5$ and $\Omega _{pT}=0.6$. $\alpha $ is set to 33.

In Fig. 3, we plot the radial stress for different boundary values ($\Omega _{pR}$ constant) and the circumferential stress for different values of $\Omega _{pT}$ ($\Omega _{bT}=0$). The peak values of the radial stress change when the boundary values change. In the case of the circumferential stress, only the peak values change as a function of $\Omega _{pT}$, while the boundary values do not change. These cases are equivalent to the simulation results shown in [3] for the radial and circumferential stresses for different growth strain values (Fig. 4 A,B in [3]). From Fig. 4 A,B in [3], we see that, as the growth strain value increases, the peak and boundary values of the radial stress increase while only the peak value of the circumferential stress increases. In [3], the behavior of SSg is demonstrated through simulations while, hereby, it is predicted by our analytical model.

**FIGURE 4.**
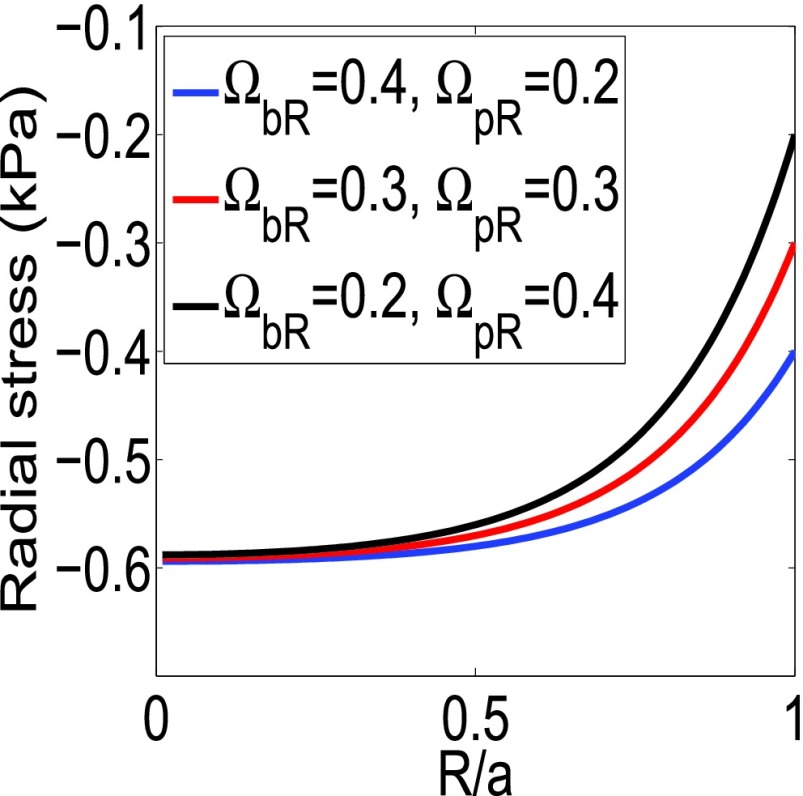
Radial SSg from the analytical model for different values of $\Omega _{bR}$ and $\Omega _{pR}$. $\alpha $ is set to 6.

In Fig. 4, we plot the radial stress for different $\Omega _{pR}$ and $\Omega _{bR}$. We see that the peak values of the radial stress remain constant but the boundary values change. These cases represent the radial stresses at different time points (day 1, day 2, etc.) for a constant growth strain in [3]. From Fig. 6A in the work of Sarntinoranont *et al.* [3], we see that, as time progresses, the boundary value of the radial stress increases, but the peak value remains the same. As the circumferential stress is always zero at the boundary, it does not change with time if the growth strain is kept constant [3]. However, it should be noted that, in practical cases, the growth strain also increases inside tumors as cancer progresses [15].

**FIGURE 5.**
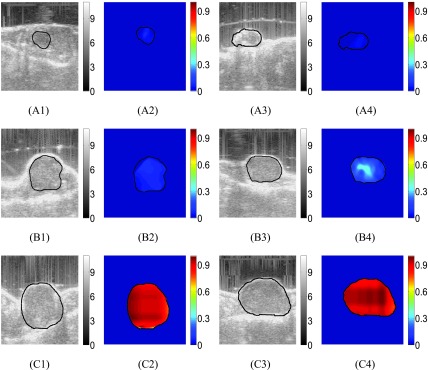
B-mode images of untreated mouse #1 at three time points (week 1, week 2, week 3) are shown in (A1), (B1) and (C1). Reconstructed SSn distributions (dimensionless) at the same time points are shown in (A2), (B2) and (C2). B-mode images of untreated mouse #2 at three time points (week 1, week 2, week 3) are shown in (A3), (B3) and (C3). Reconstructed SSn distributions at the same time points are shown in (A4), (B4) and (C4).

**FIGURE 6.**
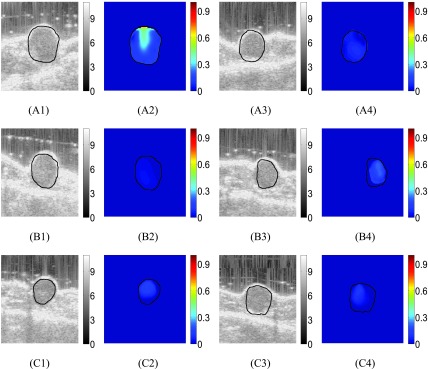
B-mode images of treated mouse #1 at three time points (week 1, week 2, week 3) are shown in (A1), (B1) and (C1). Reconstructed SSn distributions (dimensionless) at the same time points are shown in (A2), (B2) and (C2). B-mode images of treated mouse #2 at three time points (week 1, week 2, week 3) are shown in (A3), (B3) and (C3). Reconstructed SSn distributions at the same time points are shown in (A4), (B4) and (C4).

These results demonstrate that our proposed analytical model is able to model SSg in different scenarios of clinical interest for the analysis of tumors, i.e., change in growth strain, time increment etc. Our results match well with those previously reported in the literature using finite element simulations [1]–[3], [35].

### A. In Vivo Experiments

B-mode images and corresponding reconstructed SSn distributions obtained from data acquired from two untreated mice at three different time points (week 1, week 2 and week 3) are shown in Fig. 5 (A1-A4, B1-B4 and C1-C4). From the B-mode images, we see that the size of the tumor increases with time. From the SSn distributions, we see that, in the first week, SSn in both untreated tumors is very low ($\approx 0$). However, SSn increases with time, and the peak SSn is close to the highest value (1) in the third week.

B-mode images and corresponding reconstructed SSn distributions obtained from data acquired from two treated mice at three different time points (week 1, week 2 and week 3) are shown in Fig. 6 (A1-A4, B1-B4 and C1-C4). From the B-mode images, we see that, as a result of drug administration, the tumor’s size decreases or remains the same with time. From the SSn images, we see that SSn is overall low inside the tumors in all three weeks and slightly decreases in the third week.

**FIGURE 7.**
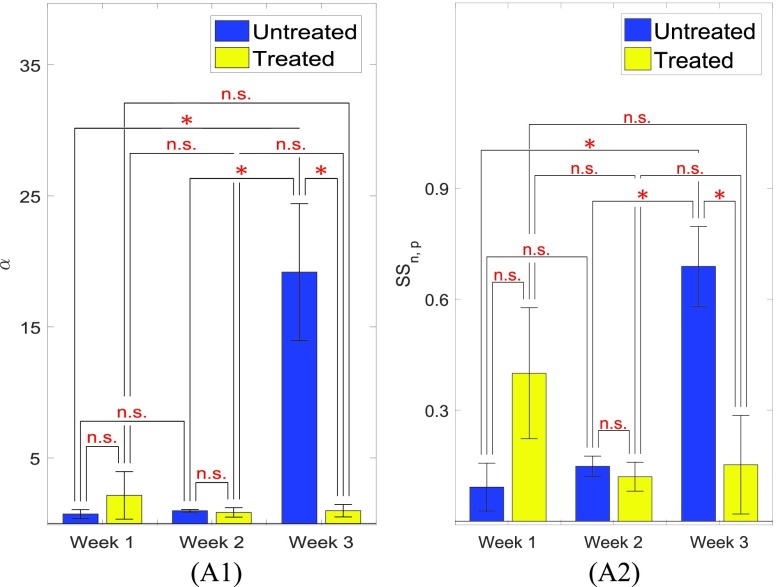
(A1) Mean $\alpha $ values for the treated and untreated mice at week 1, week 2 and week 3. (A2) Mean values of $SS_{n,p}$ for the treated and untreated mice at week 1, week 2 and week 3. n.s. means not statistically significant. One, two and three stars correspond to $p$-value less than 0.05, 0.01, 0.001, respectively.

The mean value of $\alpha $ and $SS_{n,p}$ inside the 6 treated and 6 untreated tumors are shown in Fig. 7 (A1) and (A2). From Fig. 7 (A1), we see that the value of $\alpha $ increases with time inside the untreated tumors, whereas it decreases with time in the treated tumors. Based on previous literature [25], $\alpha $ is expected to increase with cancer progression and decrease with treatment administration. Similarly to $\alpha $, the value of $SS_{n,p}$ increases with time in untreated tumors. $SS_{n,p}$ decreases inside the treated tumors from week #1 to week #2 but does not change significantly from week #2 to week #3. These results correlate well with the reported values of SSg in the literature [15].

## IV. Discussion

In this paper, we developed a method for imaging the normalized SSg inside tumors. The proposed methodology is based on an analytical model of SSg distribution inside the tumor that connects the SSg with $\alpha $ as well as the tumor’s vascular permeability and interstitial permeability. To our knowledge, our work represents the first attempt to develop an analytical model of SSg in tumors and reports the first ever generated images of the spatial distribution of SSg inside solid tumors in vivo.

Distribution of SSg in cancers is clinically informative as it directly affects cancer’s growth and metastasis while modulating the cancer microenvironment. SSg compresses the blood vessels and causes reduction in outflow of the fluid thus increasing IFP. Vessel compression also reduces the flow of the immune cells inside the tumor, induces hypoxia and acidity, which decreases the access of drugs to the tumor [14]. Thus, the development of an analytical model of SSg in cancers and in vivo determination of the normalized SSg inside tumors using a non-invasive technique can have a large impact on the analysis of growth, hypoxia, metastasis and apoptosis of tumors.

The described method to assess the stress distribution inside tumors requires knowledge of the parameter $\alpha $. The parameter $\alpha $ dictates the spatial distribution of IFP and solid stress inside a tumor and, as such, can provide valuable information for drug delivery therapies [25]. In tumors with large $\alpha $, the gradient of IFP (IFV) is small inside the tumor and high at the periphery. Therefore, most of the drug cannot reach the central portion of the tumor and accumulates at the periphery [25]. If $\alpha $ reduces, the IFV increases and the drug has a better chance to reach the central portion of the tumor. However, if $\alpha $ becomes too low (≤ 1), IFP becomes very small, IFV again reduces and the effective drug delivery gets hampered. Therefore, treatments such as vascular normalization should be administered in such a way that $\alpha $ should be close to 5, so that proper drug delivery may be achieved [25]. In tumors with low $\alpha $, it is also possible that the flux of growth factors reaching the draining lymph nodes decreases due to less fluid flow from the boundary [25], which could also inhibit lymph node lymphangiogenesis [36]. Lymph node lymphangiogenesis is thought to potentially increase the incidence of lymph node metastasis, by providing additional opportunities for the cells to enter into the lymphatic system. Therefore, a low value of $\alpha $ may reflect in an improvement of the delivery/penetration of therapeutics in tumors, alleviation of peritumoral fluid accumulation, and, at the same time, decrease the shedding of cancer cells into peritumoral fluids or surrounding tissues.

The protocol used in our poroelastography experiments is creep compression. The proven relationship between the actual solid stress and the stress developed in a poroelastography experiment is only true when the creep compression protocol is used and the assumptions mentioned in Section II are satisfied. The developed analytical model for normalized solid stress is always applicable when the inclusion’s vascular permeability is dominant over its interstitial permeability. When the interstitial permeability in the inclusion has comparable or dominant effect as the vascular permeability, the analytical model is only applicable if the interstitial permeability of the inclusion is much lower than the interstitial permeability of the background.

In poroelastography experiments, we used conventional linear array imaging for data acquisition for the following reasons. Firstly, although other advanced methods such as compound plane wave imaging have higher frame rate, they have lower spatial resolution [37], [38]. Spatial resolution is very important in our application, as we need high quality axial as well as lateral strain for our estimation of solid stress. Moreover, beam forming in these methods requires higher computation and data processing. Secondly, the applied compression is in general small in elastography and the movement of the tissue can be well-tracked using the frame rate in linear array imaging. We note that, in the future, our technique may be combined with these methods for imaging the stress distribution in applications where very high frame rates are required, such as cardiac elastography.

In our poroelastography experiments, we used one minute long data acquisitions. This data acquisition duration might be an issue in patient imaging as the breathing and motion of the patient and sonographer may introduce decorrelation noise in the data [39]. However, breathing artifacts can be minimized by using high frame rate ultrasound systems and by computing the displacements and strains from successive RF frames or RF frames separated by sufficiently short time intervals. Our strain estimation method proposed in Islam et al. [32] assumes similarity of echo amplitudes and displacement continuity while estimating the strains, which further reduces the noise due to decorrelation [40]. We have used the method described in [32] for strain estimation to compute all the results in this paper, which has been found to produce both axial and lateral strains of high quality. However, other advanced beamforming methods such as synthetic aperture [41], coherent compound plane wave imaging [42] may be used in future to further improve the strain estimation.

In the present form, our work allows estimation of the normalized SSg but does not allow quantification of the actual SSg. However, it is important to note that, in many applications, such as drug delivery and/or diagnosis and prognosis of cancers, the spatial distribution of SSn and the relative change in the value of SSg obtained from the temporal distribution of $SS_{n,p}$ along with the parameter $\alpha $ can be of great importance [14], [25]. As far as the limitations of the reported in vivo study, the number of datasets (6 treated and 6 untreated at 3 time points) used in the analysis is admittedly small for statistical conclusions. However, the observed trends in terms of mean values of the estimated parameters correlate well with results previously reported in the literature using invasive methods. In the future, it may be possible to validate some of these findings using controlled phantoms or invasive measurements.

## V. Conclusions

In this paper, we have developed a non-invasive, poroelasto-graphy-based method to image the normalized solid stress inside tumors in vivo. The proposed method is based on an analytical model of the solid stress distribution, which demonstrates that the solid stress inside the tumor has the same spatial distribution as the compression-induced stress generated in the tumor during a creep compression experiment. As the solid stress is an important component of the cancer microenvironment, the proposed technique may provide new information about cancer mechanopathology and, eventually, lead to improved cancer diagnosis and treatment methods.

## Appendix A Conversion of Stress Components From Cylindrical Coordinate System to Polar Coordinate System

The radial and circumferential SSc inside the sample (in cylindrical coordinates) can be assumed to be equal in axisymmetric conditions. Therefore, the radial and circumferential SSc components and the FPc in spherical coordinates can be determined from the SSc components and FPc in cylindrical coordinates as

\begin{align*} \sigma _{RR}(R,t)=&\sqrt {2\sigma _{rr}^{2}(\sqrt {r^{2}+z^{2}},t)+\sigma _{zz}^{2}(\sqrt {r^{2}+z^{2}},t)},\qquad \tag{8}\\ \sigma _{\theta \theta }(R,t)=&\sigma _{rr}(\sqrt {r^{2}+z^{2}},t), \tag{9}\\ p(R,t)=&p(\sqrt {r^{2}+z^{2}},t). \tag{10}\end{align*}

## Appendix B Solid Stress Created by Creep Compression and Solid Stress Induced by Growth Have the Same Spatial Distribution

We can write the following differential equation for the FPc inside a spherical tumor with IP $k$ and VP $L_{p}$ [43], [44]

\begin{equation*} \frac {1}{H_{A}}\frac {dp}{d t}+\frac {1}{H_{A}}\frac {dQ}{d t}+\chi p=k\left({\frac {d^{2}p}{dR^{2}}+\frac {2}{R}\frac {dp}{dR}}\right), \tag{11}\end{equation*}

where $Q$ is an integration constant and depends only on $t$, $H_{A}$ is the aggregate modulus of the tumor and $\chi $ is the average microfiltration coefficient. Here, $\chi =\chi _{V}+\chi _{L}$, with $\chi _{V}=\frac {L_{p}S}{V}$ and $\chi _{L}=\frac {L_{PL}S_{L}}{V_{L}}$. $L_{p}$ and $L_{pL}$ are the permeability of the capillary and the permeability of the lymphatic walls. $\frac {S}{V}$ and $\frac {S_{L}}{V_{L}}$ are the surface area to volume ratio of the capillary and lymphatic walls. Based on the values of the permeability of capillary and lymphatic walls in tumors reported in the literature, $\chi _{V}\gg \chi _{L}$ [3]. This results in $\chi \approx \chi _{V}$, and the microfiltration coefficient becomes the VP (permeability of capillary walls) multiplied by the surface area to volume ratio. It should be noted that, when IP has comparable or dominant effect with respect to VP in the inclusion, eq. (11) for FPc inside the inclusion is valid only if the IP of the inclusion is much lower than the IP of the background. On the other hand, if VP is dominant over IP in the inclusion, Eq. (11) for FPc inside the inclusion is always applicable.

At a specific time point $t=t_{0}$, to analyze only the spatial characteristics of the FPc, we can write

\begin{equation*} \frac {d^{2}p}{dR^{2}}+\frac {2}{R}\frac {dp}{dR}=\frac {\chi }{k}p-W, \tag{12}\end{equation*}

where $W=\frac {1}{H_{A} k}\frac {dp}{d t}+\frac {1}{H_{A} k}\frac {dQ}{d t}$ at $t_{0}$. Using the formula for the Laplace operator in spherical coordinates $\nabla ^{2}\,\,p=\frac {1}{R^{2}} \frac {d}{dR} \left[{R^{2} \frac {dp}{dR}}\right]=\frac {d^{2}p}{dR^{2}}+\frac {2}{R}\frac {dp}{dR}$, eq. (12) can be written as

\begin{equation*} \nabla ^{2} p=\frac {\chi }{k}p-W. \tag{13}\end{equation*}

Solving eq. (13) with boundary condition of zero FPc at the periphery of the tumor, the equation for the FPc can be written as [24], [45]

\begin{equation*} p(R)=\Psi \left({1-\frac {\sinh {\left({\alpha \frac {R}{a}}\right)}}{\frac {R}{a}\sinh {\alpha }}}\right),~\text {where}~\alpha =a \sqrt {\frac {L_{p}}{k}\frac {S}{V}}.\qquad \tag{14}\end{equation*}

Here $\Psi =\frac {W}{\alpha ^{2}}$ is a constant related to the peak FPc $P_{0}$ as $P_{0}=\Psi (1-\alpha \,\, \text {cosech} (\alpha))$ and $a$ is the radius of the tumor.

The relationship between the radial/circumferential SSc and FPc can be written as

\begin{align*} \sigma _{RR} (R,t)=&\sigma _{RR}^{a}-p(R,t), \tag{15}\\ \sigma _{\theta \theta }(R,t)=&\sigma _{\theta \theta }^{a}-p(R,t), \tag{16}\end{align*}

where $\sigma _{RR}^{a}$ and $\sigma _{\theta \theta }^{a}$ are radial and circumferential SSc at the tumor boundary and are related to the applied stress at the top of the sample. These stresses can be computed using Eshelby’s theory from the applied stress and Young’s modulus and Poisson’s ratio of the tumor [22].

We see that the radial and circumferential SSc inside the tumor have the same spatial dependence as the FPc and depends on $\alpha $, i.e., the IP and VP of the tumor. The difference between the SSc and FPc is the value at the boundary of the tumor. FPc is zero at the tumor boundary, whereas the radial and circumferential SSc have values of $\sigma _{RR}^{a}$ and $\sigma _{\theta \theta }^{a}$ at the boundary, respectively. Based on this observation and the fact that the radial/circumferential SSg, IFP and FPc in a poroelastography experiment have same spatial distributions [3], [26], we can deduce that the radial/circumferential SSc in a poroelastography experiment has same spatial distribution as the radial/circumferential SSg inside tumors.

## Appendix C Method for the Estimation of $\alpha$

Let us consider a poroelastic sample of volume V, which contains a solid phase of volume $V_{s}$ and a fluid phase of volume $V_{p}$. The total volume can be written as the sum of these two volumes.

\begin{equation*} V=V_{p}+V_{s}.\tag{17}\end{equation*}

In the above equation, any isolated pores that the fluid cannot infiltrate are considered part of the solid phase. To construct the constitutive equations, we select the volumetric strains of the sample and its fluid phase as the dynamic variables, i.e., ($\Delta V/V, \Delta V_{p}/V_{p}$). The following formulations can be developed based on the theory of the effective stresses, [46, p. 84]

\begin{equation*} \frac {\Delta V}{V}=-\frac {1}{K}(\sigma -\zeta p),\quad \frac {\Delta V_{p}}{V_{p}}=-\frac {1}{K_{p}}(\sigma -\beta p),\tag{18}\end{equation*}

where $\zeta $ and $\beta $ are the Biot’s effective stress coefficient and pore volume effective stress coefficient, respectively, $p$ is the fluid pressure and $K$ and $K_{p}$ are the drained bulk modulus and pore volume bulk modulus, respectively. It should be noted that the above relationships are a generalization of Hooke’s law.

The remaining of our analysis is based on the assumption of intrinsic incompressibility for both the solid phase and the fluid phase, for which $\zeta =1$, $\beta =1$ and $K_{p}=\infty $.

Based on this assumption, we can write

\begin{equation*} \frac {\Delta V}{V}=-\frac {1}{K}(\sigma - p),\quad \frac {\Delta V_{p}}{V_{p}}=0.\tag{19}\end{equation*}

The left hand side of the first equation is the volumetric strain. In an axisymmetric setup, the volumetric strain $\epsilon $ can be written as

\begin{equation*} \epsilon =-\frac {\Delta V}{V}=\epsilon _{zz}+2\epsilon _{rr}.\tag{20}\end{equation*}

where

\begin{equation*} \epsilon _{zz}=\frac {du_{z}}{dz},\quad \epsilon _{rr}=\frac {du_{r}}{dr}.\tag{21}\end{equation*}

The radial and axial displacements are denoted as $u_{z}$ and $u_{r}$.

Based on the above discussion, the volumetric strain and fluid pressure at any time $t$ inside a spherical tumor are related by

\begin{equation*} \epsilon (R,t)=\frac {1}{K}(\sigma -p(R,t)),\tag{22}\end{equation*}

where $K$ is the compression modulus of the tumor. At steady state ($t=\infty $), when the fluid pressure becomes zero, the volumetric strain can be written as

\begin{equation*} \epsilon (R,\infty)=\frac {1}{K}(\sigma).\tag{23}\end{equation*}

Therefore, we obtain

\begin{equation*} \epsilon (R,t)-\epsilon (R,\infty)=\frac {1}{K}(-p(R,t)).\tag{24}\end{equation*}

The fluid pressure at time $t$ can be written as

\begin{equation*} p(R,t)=-K(\epsilon (R,t)-\epsilon (R,\infty)). \tag{25}\end{equation*}

The parameter $\alpha $ can be estimated by fitting the fluid pressure data (estimated using eq. (25)) with the theoretical equation of fluid pressure (eq. (14)). The compression modulus $K=\frac {E}{3(1-\nu)}$ of the tumors and normal tissues can be computed using their Young’s modulus ($E$) and Poisson’s ratio ($\nu $).
